# Structural models of the different trimers present in the core of phycobilisomes from *Gracilaria chilensis* based on crystal structures and sequences

**DOI:** 10.1371/journal.pone.0177540

**Published:** 2017-05-18

**Authors:** Jorge Dagnino-Leone, Maximiliano Figueroa, Claudia Mella, María Alejandra Vorphal, Frédéric Kerff, Aleikar José Vásquez, Marta Bunster, José Martínez-Oyanedel

**Affiliations:** 1 Departamento de Bioquímica y Biología Molecular, Facultad de Ciencias Biológicas, Universidad de Concepción, Concepción, Chile; 2 Centre d'Ingéniérie des Protéines, Université de Liège, Liège, Belgium; University of Illinois at Urbana-Champaign, UNITED STATES

## Abstract

Phycobilisomes (PBS) are accessory light harvesting protein complexes that directionally transfer energy towards photosystems. Phycobilisomes are organized in a central core and rods radiating from it. Components of phycobilisomes in *Gracilaria chilensis* (*Gch*) are Phycobiliproteins (PBPs), Phycoerythrin (PE), and Phycocyanin (PC) in the rods, while Allophycocyanin (APC) is found in the core, and linker proteins (L). The function of such complexes depends on the structure of each component and their interaction. The core of PBS from cyanobacteria is mainly composed by cylinders of trimers of α and β subunits forming heterodimers of Allophycocyanin, and other components of the core including subunits α^II^ and β^18^. As for the linkers, Linker core (L_C_) and Linker core membrane (L_CM_) are essential for the final emission towards photoreaction centers. Since we have previously focused our studies on the rods of the PBS, in the present article we investigated the components of the core in the phycobilisome from the eukaryotic algae, *Gracilaria chilensis* and their organization into trimers. Transmission electron microscopy provided the information for a three cylinders core, while the three dimensional structure of Allophycocyanin purified from *Gch* was determined by X-ray diffraction method and the biological unit was determined as a trimer by size exclusion chromatography. The protein sequences of all the components of the core were obtained by sequencing the corresponding genes and their expression confirmed by transcriptomic analysis. These subunits have seldom been reported in red algae, but not in *Gracilaria chilensis*. The subunits not present in the crystallographic structure were modeled to build the different composition of trimers. This article proposes structural models for the different types of trimers present in the core of phycobilisomes of *Gch* as a first step towards the final model for energy transfer in this system.

## Introduction

Aquatic organisms, such as cyanobacteria, eukaryotic red alga and cryptophyta, present accessory light harvesting protein complexes called phycobilisomes (PBS) [1]. The structure of PBS allows the energy to be transferred directionally to photosystem II (PSII), thus improving the use of the 400-650nm range of wavelengths. Phycobilisomes are organized in a central core and rods radiating from it. Components of phycobilisomes in *Gracilaria chilensis* are Phycobiliproteins (PBPs): Phycoerythrin (PE), and Phycocyanin (PC) in the rods, Allophycocyanin (APC) is found in the core, and linker proteins (L) present along the whole system. PBPs are chromophorylated proteins that share a general architecture. They are organized as heterodimers (αβ), which assembly themselves into trimers or hexamers. The subunits of PBPs are mono or multi chromophorylated with linear tetrapyrrols covalently bound to cysteine residues. Efficient energy transfer is achieved through a combination of position and geometry of the chromophores and the protein environment [1–3]. In the present article, we focused on the principal component of the phycobilisomes´ core, the phycobiliprotein Allophycocyanyn or APC. The Allophycocyanin is organized as cylinders, and different numbers of these cylinders have been reported for the core of PBS including bicylindrics, tricylindrics and pentacylindrics, being the tricylindric the most common in cyanobacteria and red alga [4–6]. The tricylindrical core has been proposed to be formed by a foundation of two cylinders associated to membrane and a third cylinder is located at the top of the base forming a pyramid [7]. Four subunits have been identified for APC in the core: α, β, α^II^ and β^18^, which are codified by apcA, apcB, apcD and apcF genes, respectively [8]. Studies reported on *Synechococcus* show that subunits α^II^ and β^18^ are expressed in minor quantities, which make difficult to study the expressed proteins in native conditions [9,10]. Indeed, studies of the same nature in eukaryotic red alga are still missing.

Besides PBPs, the two linkers Core (L_C_) and core-membrane (L_CM_) have been identified in the core [1]. The L_C_ of 7.8 kDa is codified by the apcC gene. This linker is associated to APC trimers in cylinders, suggesting a specific role on stability and energy transfer [9,11–13]. The structure of L_C_ has been determined by X-ray diffraction as a complex with APC trimer in *Mastigocladus laminosus* (PDB ID: IB33) [14]. The L_CM_, codified by the apcE gene, in a tricylindrical core is a 90kDa chromophorylated linker protein that has been proposed as the final acceptor of the light harvested by the PBS, besides its role in the anchoring of the PBS to the photosynthetic membrane [9,12,13,15,16]. Different domains have been identified in the linker core-membrane; for example, the N-terminal region, called PB domain, is homologous to PBP alpha subunits, and shows 30% similarity with α subunit of APC, and also contains a phycocyanobilin chromophore. This PB domain presents an insertion of 50 residues (PB loop). The PB loop in cyanobacteria has been associated to fluorescence quenching by its interaction with the orange carotenoid protein (OCP) [17], which has not been detected in eukaryotic alga. Zhao and his group [15] produced a recombinant chromophorylated PB domain in absence of lyases, a fact that induced the authors to propose an autocatalytic binding of phycocyanobilin to L_CM_. Due to their similarity, the PB domain can replace the α subunit and form a PBβ heterodimer, which can associate itself to other αβ heterodimers to form a trimer.

Different trimers composition present in a tricylindrical core have been reported for *Synechocystis* [18] APC (α_3_β_3_), APC_1 (αα_3_β_3_Lc), APC_2 (α_2_α^II^β_3_Lc) and APC_3 (α_2_PBβ_2_β^18^). Watanabe and Ikiushi [19], in an interesting review proposed also a model for the position of each type of trimer in the three cylinders core, which in *Gracilaria chilensis* has not been assessed yet.

*Gracilaria chilensis* (Rhodophyta, Gigartinalis) (*G*.*ch*) [20] is one of the economically important red macroalga in the South Pacific. Our research on the structure and function of the PBS in this algae has been focused on understanding how the organization, *i*.*e*. composition, structure or component interactions of the PBS provides the frame for the high efficiency of the energy transfer towards PSII. Until now, the structure of PE and PC from *Gch* has been reported previously by our group [21,22], as well as information about interaction between hexamers in a rod and its role in the function of energy transfer [23]. In this article, we report i) the three cylinders nature of the core of the PBS in *Gch*, ii) the sequences of the subunits components of the core, iii) the sequence analysis and iv) the X-ray structure for Allophycocyanin from *Gch* (αβ)_3_. This structure was used to model the rest of the subunits, which may also form a functional Allophycocyanin, and thereby to build the trimers proposed for the core in cyanobacteria, as our first approach to the analysis of the contribution of both the subunits in the core and L_C_ to the energy transfer pathways in an eukaryotic red alga.

## Material and methods

### Purification of phycobilisomes

PBS were purified using the condition presented in S1 File, from 200g of intertidal *G*.*ch* collected at Coliumo Bay (36° 32’S, 072° 56’ W). The fraction containing complete PBS was identified by its spectroscopic properties (emission at 660nm after excitation at 566nm) and was used for Electron Microscopy.

*Electron microscopy*: The micrographs were obtained in a Philips Tecnai 12 Bio Twin EM (120kv) at the Pontificia Universidad Católica de Chile facility, using the methodology described [23] for complete phycobilisomes.

### The sequences of the subunits

#### Primers design

The primers were designed by sequence alignment of the genes reported in literature, using the sequences displayed on S2 File. The primers were designed manually considering the conserved sequences, the %GC, Tm and the size of the oligonucleotides. The design was verified with Oligonucleotide PrimerCheck (http://depts.washington.edu/bakerpg/primertemp/primermelttemp.html) and synthesized by IDT. The primers used were:

α subunit Sense: 5’atgagtattattactaaatcaatcgttaatgctgatgcagaagctcgg 3`

Antisense: 3’ttactgcattgcacctaatgtataatcaaaataaaatcct 5’

βsubunit

Sense: 5’atgcaagatgctattacttctgtaattaatgcagctgatgtacaagg 3’

Antisense: 3’ctagcttaaaccagaacaaatataatcaaaatatactccc5’

α^II^ subunit

Sense: 5’atgagcttagttagccaaattattttaaatgcagataatgaattaagat 3’

Antisense: 3’ ttatgacataccttgtattataaaatcaaagtagggttct 5’

β^18^

Sense: 5’atgcaagacgctattactacaattttaaatcgatatgatttaacaggaaa 3’

Antisense: 3’ ttagatatcttcttcgcttaagtttttaattatatattgaaatggttcaa 5’

#### Sequencing of the genes codifying for the subunits of Allophycocyanin

RNA was extracted from 0.1g of fresh washed and frozen alga, ground in a mortar with TRI-Reagent (Molecular Research Center) using the protocol recommended by the supplier. cDNA was obtained using Reverse Transcriptase RevertAid^TM^ kit (Fermentas) according to recommendations of suppliers. The cDNA was used for the amplification assays by PCR according to S3 File. The sequences translated to proteins were used for the resolution of the 3D structure of Allophycocyanin and for the molecular modeling of the subunits α^II^ and β^18^.

#### The sequence of the linkers associated to the CORE

The PB domain of the L_CM_:Primers were designed according to the sequences reported for L_CM_ of the red algae *Chondrus crispus* [24] and *Gracilaria tenuistipitata* [25]. The sequencing and the amplification methodology were similar to the methodology described above for α and β subunits of APC.

The L_C_: The sequence of the Linker core of *Gracilaria chilensis* was obtained from a direct analysis of the transcriptome (AN: SRX1507975) [26].

### Purification of Allophycocyanin

APC was obtained as reported in S4 File. The pure protein was characterized by its spectroscopic properties of absorption (λ^A^_max_ = 651nm) and emission (λ^E^_max_ = 660nm) and the relationship A_651_/A_280_ as purity index. This sample was used for the determination of the oligomerization state of Allophycocyanin, the spectroscopic studies and for crystallization.

### Determination of the oligomerization state

50μL of 1 mg/mL of purified APC was separated in a Superdex200 HR/30 column (Amershan Biosciences), equilibrated with 5mM phosphate buffer pH7, and associated to a Merck-Hitachi FPLC with a flux of 0.5ml/min provided by a L6210 Intelligent Pump with a L-4250 UV-Vis Detector. For the calibration, BioRad standards were used. The elution of the proteins was followed at 651 and 280nm.

### Protein crystallization

A general screening was performed in the robotic unit at Universidad de Chile. The final crystallization conditions were 2mg/mL protein concentration, 300mM NaCl, 20% v/v PEG 6000, and 100mM MES pH7.

### Determination of the three dimensional structure of APC

Crystals were diffracted at Soleil Synchrotron up to 2.3 Å resolution. The phases were obtained by molecular replacement using the data of 1KN1 [27] and the software Phenix [28]. The final model was built in Coot [29] and refined by Phenix Refine [30].

### Molecular models for the different trimers present in the core

The trimer for APC was obtained by crystallographic symmetry (P321). The different subunits were modeled with Modeller v 9.1 [31]. The templates were the α subunit of APC from the structure reported here (PDB ID: 5TJF), the PB domain in 4XXI [32], and the β subunit of 5TJF for α^II^, PB domain and β^18^, respectively. Trimers APC_1, APC_2, APC_3 were built by substitution of the corresponding subunits followed by a molecular dynamics protocol for steric rearrangement of residues. L_C_ was modelled also with Modeller using the chain O of the reported structure for *Mastigoclaudus laminosus* (PDB ID: 1B33) [14] as template, and a docking procedure was performed between the L_C_ and (αβ)_3_ or (α^II^α_2_β_3_) using ClusPro [33] without restrictions. Each final model was evaluated with PROSA [34] and RAMPAGE [35].

## Results and discussion

### The phycobilisome's core of *G*. *chilensis* is tricylindrical

**Fig 1:**
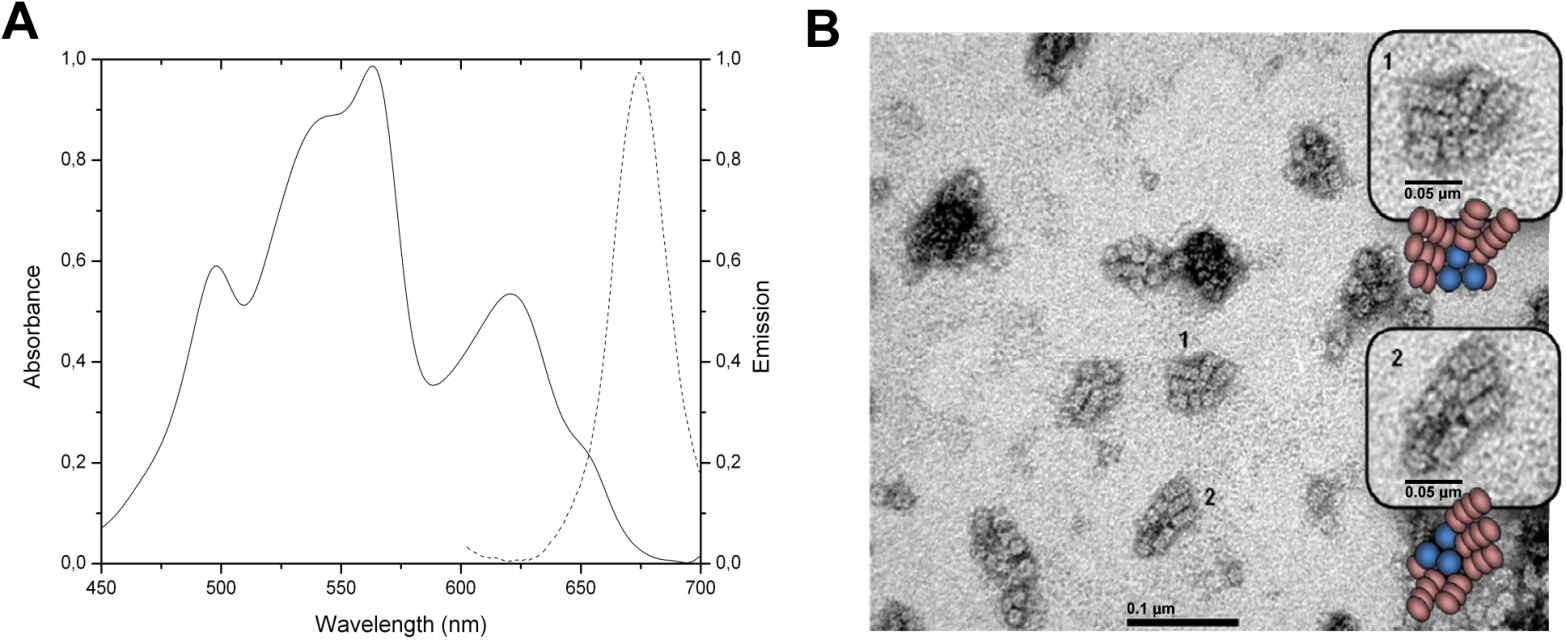
Characterization of phycobilisomes of *Gracilaria chilensis*. A) Absorption(-) and emission(..) spectra. B) Transmission electron micrograph of purified phycobilisomes.The inserts show amplified images. Schematic drawings of PBS are also shown.

The PBS enriched fractions were analyzed by electron microscopy. The absorption spectrum (Fig 1A) showed the assembly of the PBS. Upon excitation at 566nm corresponding to the absorption maximum of PE (Phycoerythrin). The only emission corresponded to the emission of APC, which means that the assembly was intact. Moreover, the energy has been transferred through the complex to emit at 660nm. The micrograph (TEM) for the PBS is shown in Fig 1B. The image clearly showed three cylinders core of phycobilisomes, which are compatible with the presence of 90kDa L_CM_, and 5–6 rods formed by the piling of 3–4 hexamers. This is the first time that a photomicrograph of a phycobilisome of *Gracilaria chilensis* is reported, and at least in shape and organization resembles the PBS from prokaryotes [2].

### Allophycocyanin is mainly present as trimer (αβ)_3_, despite the transcription of other subunits

**Fig 2:**
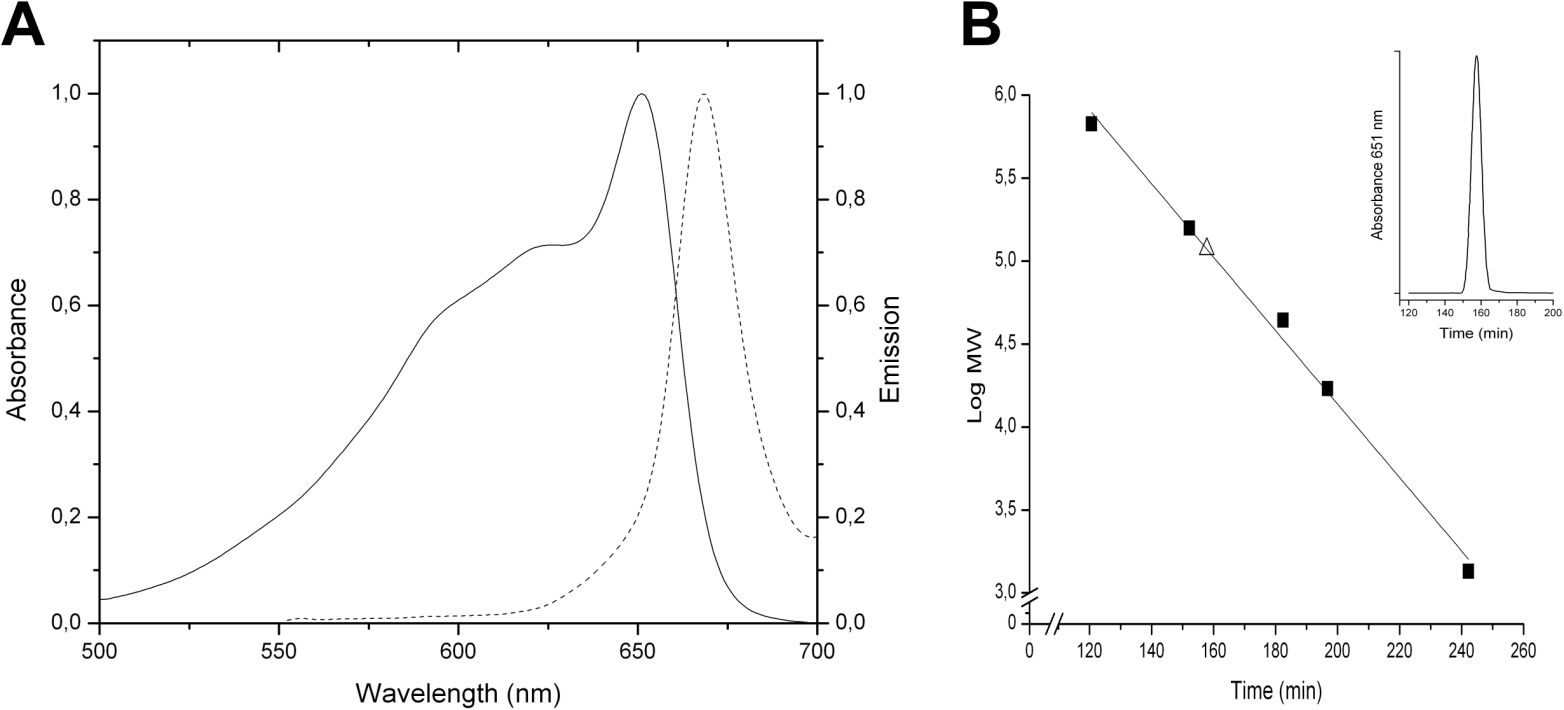
Spectroscopic characterization and oligomerization state of Allophycocyanin. A) Absorption (-) and emission (--) spectra of purified Allophycocyanin. B) Molecular sieve chromatogram; the standards and the sample are indicated.

The absorption and emission spectra of the purified samples of Allophycocyanin are shown in Fig 2A. This sample was used to determine the oligomerization state using size exclusion chromatography (Fig 2B).

**Fig 3:**
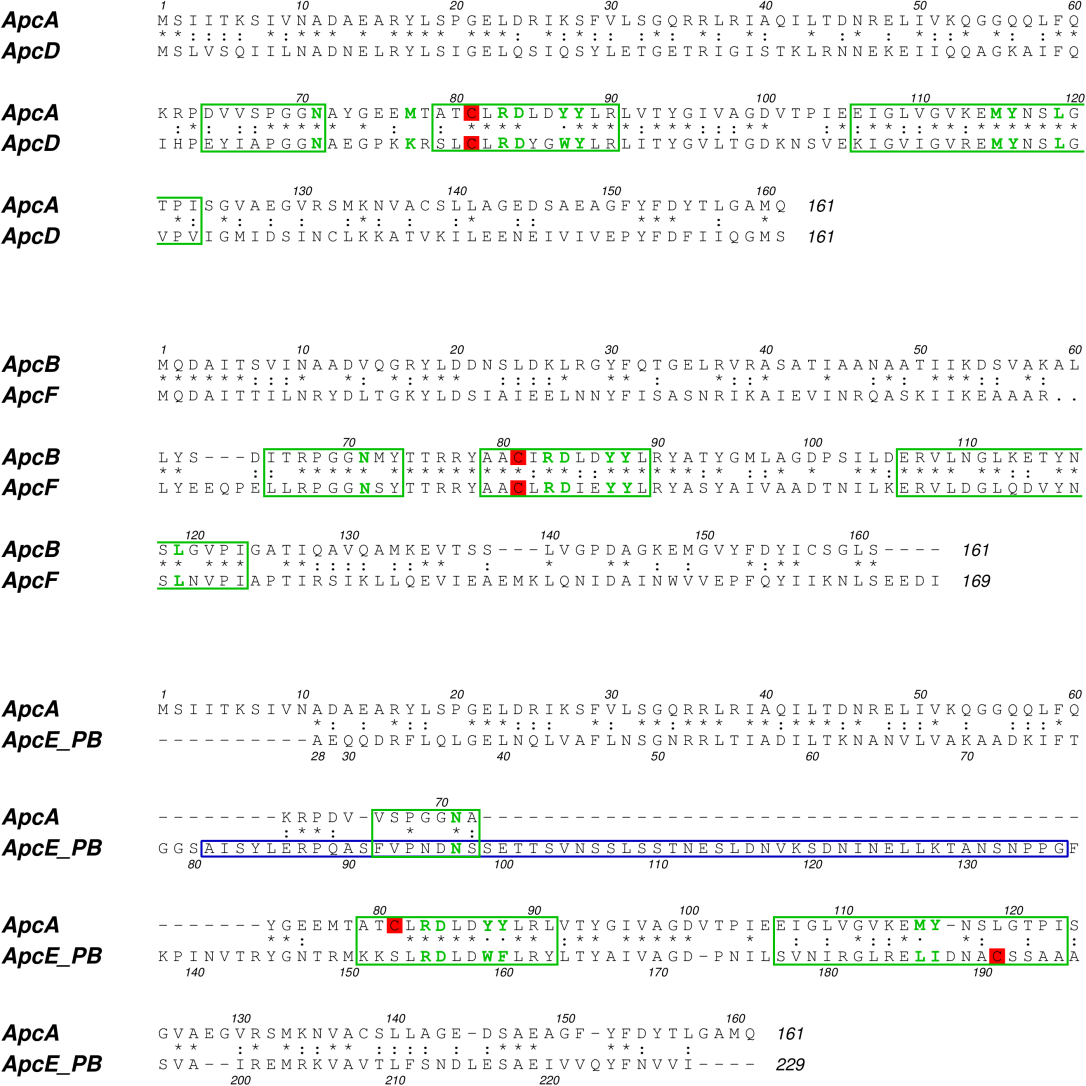
Sequence comparison between α and α^II^ (ApcA and ApcD), β and β^18^ (Apcb and ApcF) and α and the PB_domain of the L_CM_ (ApcA and ApcE). Chromophore binding region are enclosed in green rectangles, and the conserved residues are highlighted in green. The cysteine residues that bind the chromophores are shown in red background. The PB-loop sequence is enclosed in blue lines.

The result showed that Allophycocyanin from *Gracilaria chilensis* was purified in an oligomeric state (αβ)_3_. The purified APC was shown to contain only α and β subunits by mass spectrometry experiments. Studies reported in *Synechocystis* [19] and our results from the sequencing of the genes of the phycobiliproteins in the core of *Gracilaria chilensis*, revealed that they also contain α^II^ and β^18^, which are not present in the Allophycocyanin purified. Therefore, in this article the sequences of these two additional subunits are also reported (Fig 3). The sequences for apcA, apcB, apcD and apcF genes (corresponding to α, β, α^II^ and β^18^ subunits, respectively) are shown in S5 File. Fig 3 shows the amino acid sequence of the α and β subunits corresponding to the apcA and apcB genes, respectively, and comparison with sequences of their homologous subunits α^II^ and β^18^ (genes apcD and apcF, respectively). The sequence of the PB domain and the L_C_ used for this work are also displayed. These sequences were confirmed by transcriptomics analysis [26].

The analysis of the sequences showed that α and α^II^ subunits have 161 residues. They shared 41% identity and the position of the cysteines is conserved (Cys81) in both molecules. β and β^18^ subunits have 161 and 169 residues, respectively. They share 43% identity and the position of cysteines are also conserved (81 and 82, respectively). The sequence of α subunit and the fragment of the linker core-membrane used for modeling (PB domain) shared 22% identity (Fig 3), with an insertion of 55 amino acids in the PB domain. Cys81 in α subunit is replaced by Ser153 in the sequence alignment, but the chromophore is bound to Cys191 in the sequence of PB domain according to the reported recombinant structure reported (PDB ID: 4XXI) [32].

### The X-ray structure of Allophycocyanin form *G*. *chilensis* showed a high structural conservation

The purified APC was used for crystallization experiments. The statistics and the refinement data are shown in Table 1. The coordinates have been deposited at the Protein Data Bank under the accession code 5TJF.

**Table 1:** Information on the data collection and refinement for the determination of the three dimensional structure of Allophycocyanin from *Gracilaria chilensis*.

Data collection	Allophycocyanin
Space Group	P321
*Cell Dimensions*	
a, b, c (Å)	97.41, 97.41, 63.83
α, β, γ (°)	90, 90, 120
Resolution range (Å)	42.18–2.3 (2.38–2.30)
Rmerge	0.1303 (0.3568)
*I* / σ*I*	6.18 (2.16)
Completeness (%)	97 (97)
Redundancy	2.9(2.6)
*Refinement*	
Resolution (Å)	2.3
Nº reflections/Nº reflections used	84722(7757)/29317(2937)
Rwork / Rfree	25.70/28.65
Nº atoms	3035
Protein	2422
Ligand/ion	135
Water	478
B-factors	31.75
Protein	32.01
Ligand	29.65
Water	31.04
*r*.*m*.*s*. *deviation*	
Bond lengths (Å)	0.004
Bond angles (°)	1.56

The values in parenthesis correspond to the last resolution shell.

**Fig 4:**
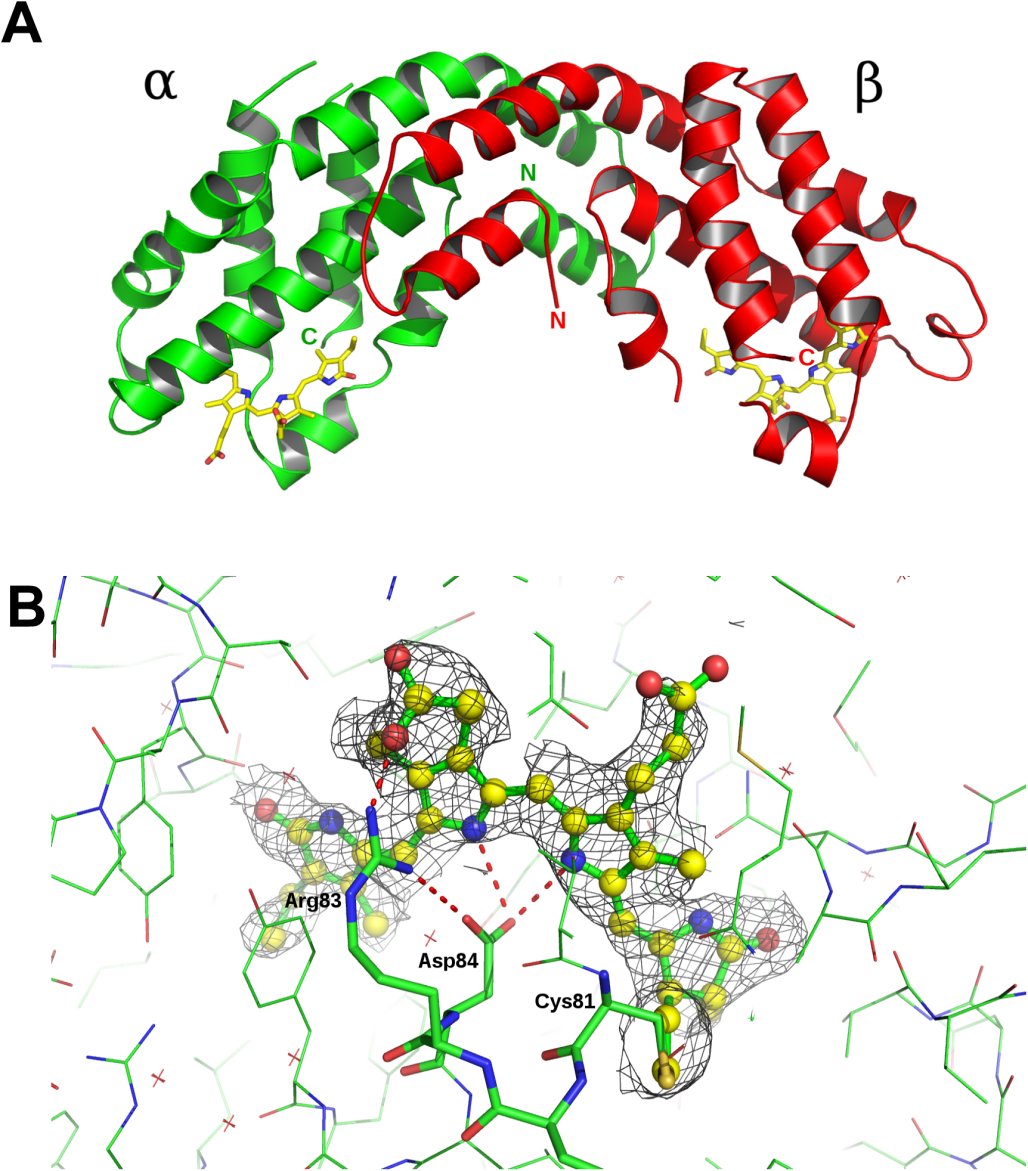
Crystallographic structure of Allophycocyanin from *Gracilaria chilensis*. A) Ribbon representation of the asymmetric unit, the heterodimer. B) A section of the |2F-Fo| omit electron density map is shown for phycocyanobilin in α subunit. The residues interacting with the chromophore are also shown.

The crystal structure of Allophycocyanin from *G*. *chilensis* was determined in the space group P321 with one heterodimer αβ per asymmetric unit using molecular replacement. The final structure was refined to 2.3Å resolution with a final R-work and R-free of 25.7 and 28.69 respectively (Table 1). The resolution allowed the chromophores to be clearly defined in the electron density map (|2Fo-Fc|) (Fig 4B).

The structure of the (αβ heterodimer is shown in Fig 4A. Indeed, α and β subunits contained one phycocyanobilin bound to cysteine 81 and are stabilized by Asn71, Arg83 and Asp84 in α subunit, and by methyl-Asn71 (Asm71) and Asp84 in β subunit. The biological unit is a trimer of heterodimers as it was determined by size exclusion chromatography. The structure reported in this article is very similar to the structures of APC previously reported, 1B33 that contains the Lc [14] and 1KN1 from the rhodophyta *Porphyra yezoensis* [27].

### The environment of the chromophore phycocyanobilin is highly conserved among different subunits

**Fig 5:**
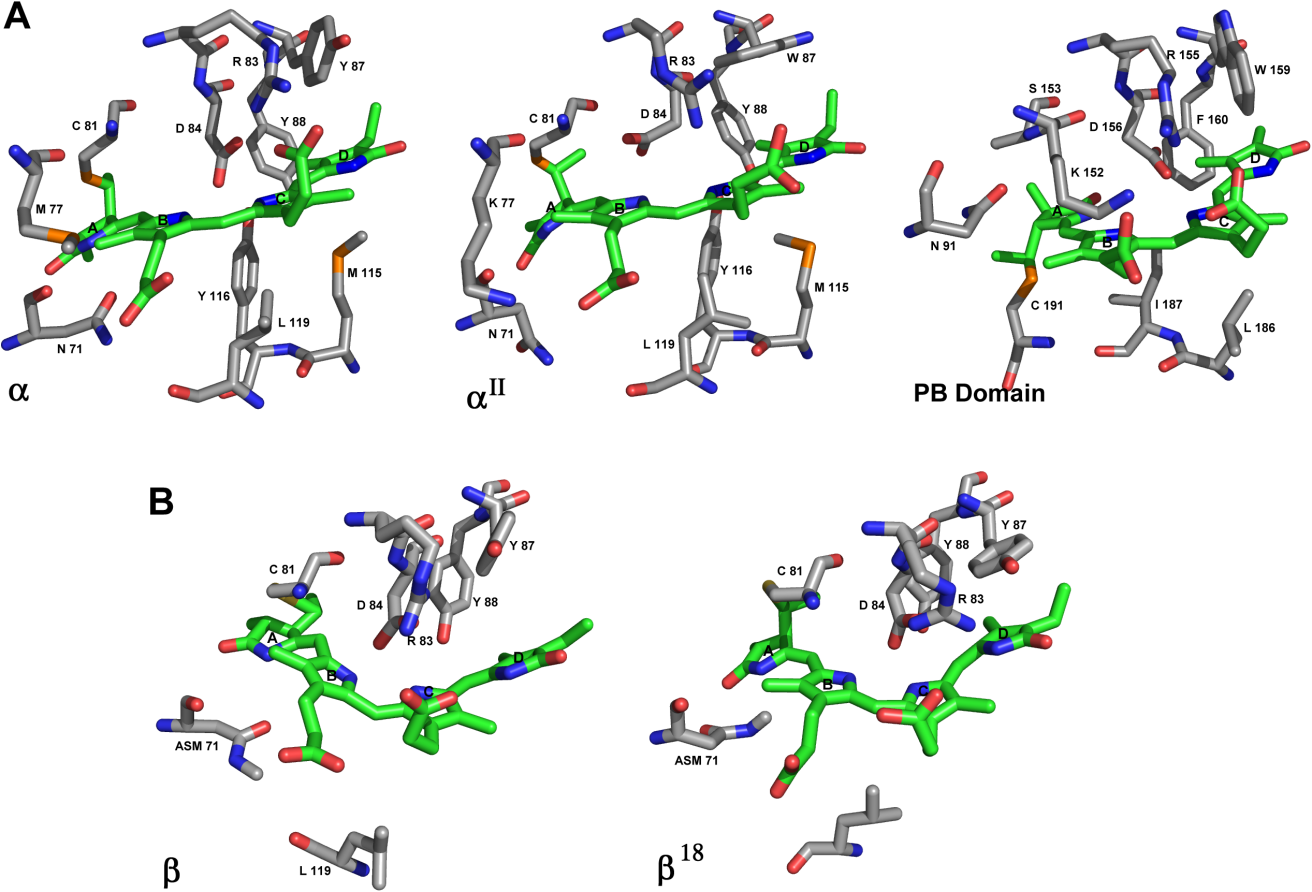
Binding sites of phycocynobilin. A) Sticks representation of binding site of phycocyanobilin in α subunit of 5TJF (this paper), α^II^ and the PB domain in APC_3. The chromophores are represented in green. B) Sticks representation of β subunit in 5TJF and β^18^ in APC_3. The chromophores are also in green.

Subunits α^II^, β^18^ and PB domain were modeled and analyzed. A comparison among subunits in APC of *M*. *laminosus* [14], *P*. *yezoensis* [27], *G*. *chilensis* (PDB IDs 1B33, 1KN1, and 5TJF (this paper), respectively) and the α^II^ model showed that the residues interacting with the phycocyanobilin chromophore are highly conserved (S1 Table). There is also high degree of conservation for the conformation of the chromophore (ASA) [36, 37], which is confirmed by the distances between rings A and D in the phycocyanobilin (Fig 5A and S1 Table). Instead the PB domain presents a SSA conformation that could account for the different spectroscopic characteristics of the L_CM_. A comparison between the β and β^18^ subunit is also shown in Fig 5B. In general, there are not important differences between β and β^18^ subunits and between α and α^II^ subunits as it was expected by the sequence identity.

The same comparison was performed between α subunit of 5TJF, the structure reported for the PB domain from *Nostoc sp*. (PDB ID: 4XXI) [32], and the model for the PB domain in *Gracilaria chilensis*. The PB domain can replace an α subunit in a trimer of heterodimers. Nevertheless, there are obvious differences, such as the insertion of 55 residues between residues 81–136 in the PB domain; 58% of this sequence was recognized as intrinsically disordered by Disprot [38] and also contains 12 Ser, 4 Thr and 1 Tyr, all hydroxylated residues. Four of these residues (Ser98, Ser99, Thr101 and Thr102) were recognized as phosphorylation sites by PROSITE and one of them (Thr101) in a motif recognized by protein kinase C. In prokaryote, this peptide segment has been described as an interaction region for the Orange Carotenoid Protein (OCP), which has not been reported in eukaryotes yet, and also a site for regulation by phosphorylation [39], fact that has not been tested in eukaryotic red algae for potential significance.

Attention is drown to the fact that the L_CM_ and its chromophore have been proposed to be responsible for the final transfer of energy towards chlorophylls in Photosystems. In this regard, it was important to analyze the differences in the binding site of phycocyanobilins in α subunit, the modeled PB domain and the structure reported at the PDB ID: 4XXI [32]. The position of Cys81 in the alignment (Fig 3), which binds the PCB in all the other α subunits, is occupied by Ser153 in the PB domain. The Cys191 is the residue that binds to the phycocyanobilin. In our model, the chromophore was stabilized by Asp156, Arg155, Trp159, and Phe160 (S1 Table). Besides the difference in the cysteine residue, it is possible to see minor differences in the stabilization of the ligand (Fig 5A and S1 Table). Considering the binding site and the conformation of the chromophore, the phycocyanobilin in the PB domain has a SSA conformation instead of ASA for the chromophore in theα subunit [36, 37]. This conformation is the same in the crystal structure of PB domain from *Nostoc* sp. (PDB ID: 4XXI) [32]. The absorption spectrum, calculated for phycocyanobilin in the model of the PB domain of *Gch*, using Gaussian 09[40] with a ZINDO-MN semiempirical approach [40], gave the λ_max_^Abs^ = 661nm, which agreed with the maximum absorption of the PB domain reported for *Nostoc sp*. Other relevant difference is that Cys 81 of α subunit binds from the top of PCB, while Cys 165 binds from the bottom in the PB domain (See Fig 5). This difference may be related to the fact that PCB needs a specific cysteine lyase to be bound to Cys 81 in α subunit. However, it has been reported that for L_CM_, the process of binding the chromophore would be autocatalytic [15]. It has been also reported a mechanism for the addition of PCB to Cys 155 in Phycocyanin in cyanobacteria. It involves the presence of an aspartic acid participating in the formation of an acylimmonium cation on ring A of the phycocyanobilin. After that, the addition reaction to cysteine is produced with the assistance of a tyrosine residue. The authors also report that after the addition, a conformational change is produced in the chromophore. The PB domain model from *Gch* locates also a tyrosine and an aspartic acid in the vicinity of the phycocyanobilin; however the position of the functional groups seems to be located too far in the binding state. It is possible that before the reaction to occur and the positioning of the chromophores, these residues could be part of the catalysis, and after the binding their location changes to the final position in the molecule.

### The crystal structure of Allophycocyanin allowed the building of the different type of trimers present in the core

**Fig 6:**
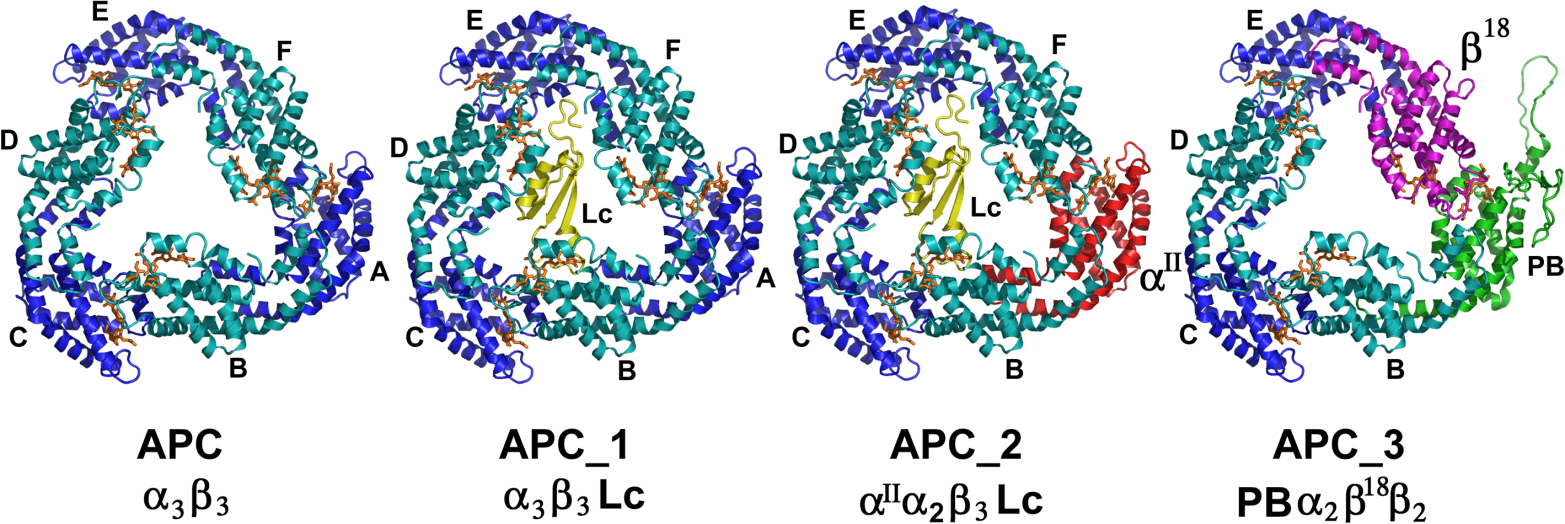
Structural models proposed for each trimer of Allophycocyanin. A) Schematic representation of the different composition trimers. B) Structure of APC, APC_1, APC_2, and APC_3.

Fig 6 shows the models created for APC, APC_1, APC_2 and APC_3 using the structure of APC reported here, with the corresponding substitution of subunits as is shown.

Each trimer was analyzed considering the number and type of interactions among subunits, the interaction surfaces on each trimer as a whole and considering the substitutions. (The data is shown in S2 Table). A comparison between trimers as follows:

1. *APC/APC_1*: The presence of the Lc (Chain G) did not affect the interaction surface (aprox.1450 Å^2^) between subunits, yet the number of hydrogen bonds between heterodimers decreases. The Lc interacted mainly with the β (chains B, D and F) subunits by hydrogen bonding.
2. *APC_1/APC_2*: Both trimers contained the Lc, but APC_2 has the substitution of α by α^II^ (Chain A). The interactions of Lc in both oligomers were very similar with β chains, especially with chain D.
3. *APC/APC_3*: The substitution of chain A (α) by PB domain and chain F (β) by β^18^ produced a trimer that contained these two special subunits in neighbor heterodimers. The αβ heterodimers conserved the interactions (H-bonds/salt bridges), but less salt bridges were observed in heterodimers with the substitutions. The interaction surfaces in both trimers are similar (aprox 1450Å^2^), except for the lower value measured for the interaction between subunits F and E (1154 Å^2^)

The structural analysis of the models proposed in this article allowed to arrange them face to face to form hexamers, which interact back to back with other hexamers to organize the cylinders present in the core as it was proposed for *Synechococcus* [41].

The experimental data available so far, for the subunit composition of trimers proposed in this article, indicated that L_CM_ (PB_domain) and β^18^ were not in the same heterotrimer with α^II^ [41]. According to spectroscopic data obtained by Kuzminov *et al* [42], β^18^ should transfer energy to the LC_M_, a fact that could be accomplished only if they are in the same trimer but neighboring heterodimers. Finally, they propose that β^18^ is a mediator in the energy transfer towards the final emitter, with the chromophore present in the L_CM_ (PB_domain).

The modeling of the 55 extra residues (PB_loop) and the analysis of the sequence (Fig 3) indicated a very disordered region that is probably interacting with other proteins. Cross-linking experiments involving cyanobacterial phycobilisomes [43] do not provide information on the possible interactions of this segment of PB domain.

The model for the Linker core fits very well the space in the center of APC; clearly, this small molecule contributes to give asymmetry to allophycocyanin. It has been reported that the L_C_ modifies the spectroscopic properties of the PCB in APC_1 and APC_2. According to our results and those from Reuter *et al* [14], in the trimeric state, one explanation is the different environment of the chromophore upon binding of L_C_.

### Future perspectives

Our work on the structure of the phycobilisome components of an eukaryotic algae such as *Gracilaria chilensis*, including the study of the components of its core in this article, pretends to fill a gap produced by the enormous amount of information published in prokaryotes, especially *Synechocysti*s and the small amount of information produced about this system in eukaryotic organisms. From the beginning, the purpose of our study on Phycobilisomes was to understand how the structure of this super complex supported the high efficiency of the energy transfer towards the photosystems. In this article, we are reporting new pieces of the big puzzle.

## Supporting information

S1 File Methodology for phycobilisomes purification. (DOCX)

S2 File Species and sequences used to design the primers for PCR. (DOCX)

S3 File Methodology for amplification assays. (DOCX)

S4 File Methodology for Allophycocyanin purification. (DOCX)

S5 File Sequence of the genes codifying for the proteins present in the core of a phycobilisome of *Gracilaria chilensis*. (DOCX)

S1 Table Comparison of the PCB binding sites for α subunits. (DOCX)

S2 Table Interface characteristics of different trimers of Allophycocyanin. Interface area, numbers of hydrogen bonds and salt bridges between subunits in different trimers.
(DOCX)
