# Supplementary material for: Structural models of the different trimers present in the core of phycobilisomes from Gracilaria chilensis based on crystal structures and sequences
Source: PLoS One. 2017 May 18;12(5):e0177540. doi: 10.1371/journal.pone.0177540 (PMC5436742; doi:10.1371/journal.pone.0177540)
Supplement: S1 File — (DOCX) [file pone.0177540.s001.docx]

**S1**

Purification of phycobilisomes. The alga was washed, frozen in liquid nitrogen and pulverized in a mortar. The powder was incubated with the *lysis phosphate buffer*(1M KH_2_PO_4_ / K_2_HPO_4_ pH 7.5) supplemented with 2.0 % v/v tritón X-100 and a cocktail of protease inhibitors EDTA free III (Calbiochem), during two hours at room temperature in the dark. The suspension was filtered (6 layers of cheesecloth), centrifuged (27000g for 20min) and the supernatant was ultracentrifuged (122000g for 3h at 4ºC). The pellet was suspended in 250ml of 0.9M KH_2_PO_4_ / K_2_HPO_4_) pH 7.0 buffer, and stirred overnight at room temperature in the dark. PBS were separated by ultracentrifugation (137000g, 3h at 4ºC), in a discontinuous sucrose gradient (2,0 M – 1,0 M – 0,5 M -0,25 M) in 0.9 M phosphate buffer pH 7.0.
