# Supplementary material for: Structural models of the different trimers present in the core of phycobilisomes from Gracilaria chilensis based on crystal structures and sequences
Source: PLoS One. 2017 May 18;12(5):e0177540. doi: 10.1371/journal.pone.0177540 (PMC5436742; doi:10.1371/journal.pone.0177540)
Supplement: S2 File — (DOCX) [file pone.0177540.s002.docx]

**S2**

Sequences used to design the primers used for PCR sequencing of the alfa and beta subunits: *Porphyra purpurea* (GeneID: 809891),

*Porphyra yezoensis* (GeneID: 3978963),

*Gracilaria tenuistipitata var lui* (GeneID: 2944001),

*Cyanidium caldarium* (GeneID: 800211)

*Cyanidioschyzon merolae strain 10D*(Gene ID: 845162),

*Cyanophora paradoxa* (Gene ID: 801537) ,

*Thermosynechococcus elongatus BP-1*( GeneID: 1011365) ,

*Gloeobacter violaceus PCC7421*( GeneID: 2598914) ,

*Nostoc sp* PCC7120 (GeneID: 1103618),

*Synechococcus sp PCC* 7002(GeneID: 6055786),

*Microcystis aeruginosa* NIES483(GeneID: 5861825),

*Cyanothece sp* (GeneID: 6169966),

*Synechocystis sp* PCC6803 (GeneID:954530),

*Synechococcus sp* WH8102 1 (GeneID:1730902), and

*Paulinella chromatophora* (GeneID:6481367).
