# Supplementary material for: Structural models of the different trimers present in the core of phycobilisomes from Gracilaria chilensis based on crystal structures and sequences
Source: PLoS One. 2017 May 18;12(5):e0177540. doi: 10.1371/journal.pone.0177540 (PMC5436742; doi:10.1371/journal.pone.0177540)
Supplement: S3 File — (DOCX) [file pone.0177540.s003.docx]

**S3**

*Amplification assays:* The reaction mix was10μL buffer, 3μL 10mM dNTP, 2μL 20μM primer Sense, 2μL 20μM primer Antisense, 1μL 1U/μL Kappa Hi-Fi, 3μL 40ng/μL template cDNA, 29μL H_2_O nuclease free in a total volume of 50μL. The protocol involved 35 cycles of 2 min at 95ºC, 30s at 98ºC, 30s at 48ºC, 45s at 72ºC, and 2min at 72ºC. The PCR products were maintained at 4ºC. Agarose gels electrophoresis was used to test the quality and to visualize the PCR products. PCR products were recovered from the gel using *Zymoclean Gel DNA Recovery* kit from Zymo Research, they were quantifies and sent for sequencing at the ABI PRISM 3100 Genetic Analyzer service of the Departamento de Ecología de la Pontificia Universidad Católica de Chile.
