# Supplementary material for: Structural models of the different trimers present in the core of phycobilisomes from Gracilaria chilensis based on crystal structures and sequences
Source: PLoS One. 2017 May 18;12(5):e0177540. doi: 10.1371/journal.pone.0177540 (PMC5436742; doi:10.1371/journal.pone.0177540)
Supplement: S4 File — (DOCX) [file pone.0177540.s004.docx]

**S4**

Purification of Allophycocyanin. Upon an extraction of phycobiliproteins from intertidal *G.ch* collected at Coliumo Bay (36° 32’S, 072° 56’ W), in 5mM Phosphate buffer pH8, followed by successive Ammonium Sulfate precipitations at 30% and 60% saturation. The precipitated protein was suspended in 50mM phosphate buffer pH7, it was dialyzed to remove salts and it was separated by ionic exchange chromatography (IEX DEAE FF 16/10 column), equilibrated with the same buffer, in a FPLC Merck-Hitachi chromatographer) and eluted with 1M KCl gradient(85%-15%); fractions with absorption at 651nm (λ^A^_max_ for APC) were collected, concentrated and further purified. The complete separation of the Allophycocyanin was accomplished after the elimination of contaminant Phycoerythrin using a hydroxylapatite column loaded in 5mM Phosphate buffer pH8 and eluted with 300 mM KCl. A final step of purification by molecular exclusion ( superdex G200) was used to eliminate oligomers of low molecular weight.
