# Supplementary material for: Structural models of the different trimers present in the core of phycobilisomes from Gracilaria chilensis based on crystal structures and sequences
Source: PLoS One. 2017 May 18;12(5):e0177540. doi: 10.1371/journal.pone.0177540 (PMC5436742; doi:10.1371/journal.pone.0177540)
Supplement: S5 File — (DOCX) [file pone.0177540.s005.docx]

**S5**

The sequence of the genes codifying for the proteins in the core of a phycobilisome of *Gracilaria chilensis*.

**>apcA**

atgagtattattactaaatcaattgttaatgcagatgcagaagctagatatttaagtccaggagaattagacagaattaaaagttttgttttatctggtcaaagacgcttaaggatagcacaaattttaacagacaaccgtgaactaattgtaaagcaaggtggtcaacagttatttcagaagaggccagatgtggtctcgcctggcggtaatgcttacggggaagaaatgactgcaacatgtttgagggatttagattattatttaagattagtgacttacggtatagtagctggtgatgtaactccaatagaagaaataggtttagtgggagttaaagaaatgtacaactctctaggtactcctatctctggagttgctgagggtgtacgttccatgaagaatgttgcttgttctttgcttgctggagaagattctgcagaggcgggtttttattttgattatacattaggcgcaatgcagtaa

**>apcB**

atgcaagatgctattacttctgtaattaatgcagctgatgtacaaggtagatatttagacgataattcattagataagttaagaggttattttcaaacaggcgaattaagagttagagcttcagctactatagcagctaatgcagcaacaattattaaagattctgtggctaaagctttattatattcagatataactaggccaggtggtaatatgtatactacaagaagatatgctgcttgtatacgtgatttagattactatcttcgttatgctacttatggaatgctagcaggcgatccatctattctagatgagcgtgttttaaatggcttaaaagaaacatataattcattaggtgtgcctattggtgctactatccaggctgtacaagccatgaaagaagttacatctagtttagtaggtccagatgcaggtaaagagatgggagtatattttgattatatttgttctggtttaagctag

**>apcC**

cgttccttcaaggtcacctatgtgcaaccgagcgaaaccagactctttacattccgcgagttgcaaaatgtttacactaccaaggttgtgccgttttcatcttggtatgctgaacagcagcgtatccaaaagatgggcggtcgtattctgaatgttgagctctcttcgggcggccagatgaggtcagtgggaaacacctaa

**>apcD**

atgagcttagttagccaaattattttaaatgcagataatgaattaagatatcttagcattggtgaattacagtcaattcaaagctatctcgaaacaggagaaacacgtattggcataagcactaaactaagaaataacgaaaaggaaattatacaacaagctggtaaagccatttttcaaattcatccagaatatatagctcctggaggtaatgcagaagggcctaaaaaaagatctttatgtcttcgagattatggatggtatttacgcctcataacgtatggtgttctaactggagataagaattccgtcgaaaaaattggtgttattggagtaagagaaatgtataactcattaggagtacctgttataggtatgattgatagtatcaattgtttaaaaaaagcaactgtgaaaattttagaagaaaatgagatagtaattgtagaaccctactttgattttataatacaaggtatgtcataa

**>apcE_PB**

gcagaacaacaagatagatttttacaattaggtgaattgagtgaattagtctctttttttaattccggtaataaaagattagagattgcacaaattttatctaaaaatgccaatattttagttgctaaggcgtctgataaaatttttgttggtggttcagctatttcatatttagaaagaccccaagcatcatttttagatactactaattcgagtaatatgtcggagatacaagaactatctggtaatactcaaaataattttttgcaaggtttttcatctacatttaattctagtgattcattgccaccaggttttaagccaattaatgttactcgttacggtaatactcgcatgaaaaagtctttacgagatttagattggtttttaagatatcttacatatgcaattgttgctggtgatcctaatattttatcagtaaatattcgtggtctcagagaattaattgataatgcatgctctagtgctgcagctagtgttgcaatacgcgagatgcgtaaagtagctgtaactttattcagtaatgacctagaatcagcagaaattgttgtgcaatattttaatgttgtgatc

**>apcF**

atgcaagacgctattactacaattttaaatcgatatgatttaacaggaaaatatttagatagtatagcaatagaagaattgaataattattttattagtgcttcaaatagaattaaagccatagaagttattaaccgtcaagcttctaaaataattaaagaggctgctgcacgactatatgaagaacaacctgaacttttaaggcctggcgggaattcctatacaactagaagatatgctgcttgcttacgagatattgaatactacttaagatatgccagttatgcaatagttgcagcagatactaatattttaaaagaacgggtattggatggattacaagatgtatataattcactgaatgttcctattgcacctactattagaagcattaagcttttacaagaagtaatagaagcagaaatgaaattacaaaatattgatgcaataaattgggttgttgaaccatttcaatatataattaaaaacttaagcgaagaagatatctaa

**apcA, apcB, apcC, apcD, apcF, and apcE_PB are the sequences codifying for α, β, Linker core, α^II^, β^18^, and the PB domain of the Linker core membrane respectively.

*apcA, apcB, apcC sequences were also confirmed by RNA seq; apcE was also confirmed by sequencing.
