# Supplementary material for: Structural models of the different trimers present in the core of phycobilisomes from Gracilaria chilensis based on crystal structures and sequences
Source: PLoS One. 2017 May 18;12(5):e0177540. doi: 10.1371/journal.pone.0177540 (PMC5436742; doi:10.1371/journal.pone.0177540)
Supplement: S1 Table — (DOCX) [file pone.0177540.s006.docx]

**S6**

Table 1. Comparison of the PCB binding sites for α subunits.

1B33 1KN1 5TJF α^II^ PB domain 4XXI

(15) (30) * * & * & (36)

Bound to Cysteines 80 84 81 81 191 196

Conformation of PCB ^(50,51)^ ASA ASA ASA ASA SSA SSA

Distances (Å)

CO^A^-CO^D^ 15.99 15.71 16.13 17.71 9.74 9.74

N^A^ - N^D^ 11.64 11.80 11.70 11.80 8.20 8.20

Residues conserved N 70 N 72 N 71 N 71 N 91 N154

R 82 R 86 R 83 R 83 R155 R160

D 83 D 87 D 84 D 84 D156 D161

Y 85 Y 90 Y 87 W 87 W159 W164

Y 86 Y 91 Y 88 Y 88 F160 F165

M114 M118 M115 M115 L186 L188

Y115 Y119 Y116 Y116 I187 I191

* This work

& Molecular modelling
