# Supplementary material for: Structural models of the different trimers present in the core of phycobilisomes from Gracilaria chilensis based on crystal structures and sequences
Source: PLoS One. 2017 May 18;12(5):e0177540. doi: 10.1371/journal.pone.0177540 (PMC5436742; doi:10.1371/journal.pone.0177540)
Supplement: S2 Table — Interface area, numbers of hydrogen bonds and salt bridges between subunits in different trimers. (DOCX) [file pone.0177540.s007.docx]

**S7**

Table 1. Interface characterization of different trimers of Allophycocyanin.

**Area in A^2^ Number of Hydrogen bonds Number of Salt bridges**


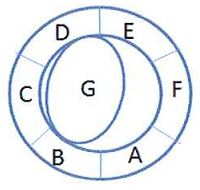
Chain\ TRIMER APC APC_1 APC_2 APC_3 APC APC_1 APC_2 APC_3 APC APC_1 APC_2 APC_3

BA 1481 1472 1471 1500 16 11 10 11 8 7 4 7

FE 1466 1467 1418 1154 14 13 7 9 5 6 5 2

DC 1410 1420 1468 1454 12 12 15 14 6 6 5 4

FA 592 469 495 523 11 7 6 7 2 5 0 2

DE 499 494 522 616 7 6 6 11 2 1 0 8

BC 401 469 428 492 4 5 8 6 1 2 3 4

GD 638 557 11 8 4 4

GB 563 480 8 1 0 0

GF 367 457 3 7 5 2

-----------------------------------------------------------------------------------------------------------------------------------------------------------------------

*The data were obtained with PISA (www.ebi.ac.uk/pdbe/pisa**)**
